# Photobiomodulation: An Effective Approach to Enhance Proliferation and Differentiation of Adipose-Derived Stem Cells into Osteoblasts

**DOI:** 10.1155/2021/8843179

**Published:** 2021-03-22

**Authors:** Daniella Da Silva, Anine Crous, Heidi Abrahamse

**Affiliations:** Laser Research Centre, Faculty of Health Sciences, University of Johannesburg, P.O. Box 17011, Doornfontein, Johannesburg, South Africa 2028

## Abstract

Osteoporosis is regarded as the most common chronic metabolic bone condition in humans. In osteoporosis, bone mesenchymal stem cells (MSCs) have reduced cellular function. Regenerative medicine using adipose-derived stem cell (ADSC) transplantation can promote the growth and strength of new bones, improve bone stability, and reduce the risk of fractures. Various methods have been attempted to differentiate ADSCs to functioning specialized cells for prospective clinical application. However, commonly used therapies have resulted in damage to the donor site and morbidity, immune reactions, carcinogenic generation, and postoperative difficulties. Photobiomodulation (PBM) improves ADSC differentiation and proliferation along with reducing clinical difficulties such as treatment failures to common drug therapies and late initiation of treatment. PBM is a noninvasive, nonthermal treatment that encourages cells to produce more energy and to undergo self-repair by using visible green and red and invisible near-infrared (NIR) radiation. The use of PBM for ADSC proliferation and differentiation has been widely studied with multiple outcomes observed due to laser fluence and wavelength dependence. In this article, the potential for differentiating ADSCs into osteoblasts and the various methods used, including biological induction, chemical induction, and PBM, will be addressed. Likewise, the optimal laser parameters that could improve the proliferation and differentiation of ADSC, translating into clinical success, will be commented on.

## 1. Introduction

Osteoporosis is a painful, chronic bone disease in humans, and its incidence is increasing globally [1]. Osteoporosis is characterized by the overall decrease in bone facets which brings about the fragility of bones and a highly probable risk of fractures [2]. At present, while therapy is still successful to some degree, there is a large discrepancy in the care of this disease. Regenerative medicine is considered a possible solution by the medical science world. The aim is to heal and treat diseased, impaired, or age-related tissue complications [3]. Currently, standing at the frontlines of regenerative medicine is stem cell therapy, due to the ability of stem cells to self-renew and differentiate into various cell types [4]. Stem cells have the unique ability of branching into numerous cell types, one of which is adipose-derived stem cells (ADSCs) [5]. ADSCs have the ability of differentiating into a specialized cell line through the use of various growth factors and physical factors [6]. PBM is understood to enhance the proliferation and differentiation of cells [7]. However, the numerous but unestablished methodologies to this technique must still be developed and pursued for a recognizable difference in stem cell therapy. This review focused on research outcomes of successful ADSC differentiation into osteogenic cell lineages. The selected thirty-one result articles summarized in the tables provided involved the use of chemical and biological growth factor inducers, PBM, and their combination for the purposes of cell differentiation. This is to reveal the potential of this particular regenerative therapy as a worthwhile *in vitro* pursuit for successful clinical studies and produce an enhanced form of treatment for osteoporosis.

## 2. Literature Review

### 2.1. Osteoporosis

Osteoporosis is a life-long skeletal disease [8] that is increasingly becoming a global epidemic [9]. Osteoporosis is defined as a decrease in bone mass, a decrease in bone density, and an overall deterioration of bone [2, 10]. This results in the weakening of bones which raises the incidence of fracture risks [10]. The World Health Organization (WHO) criteria define osteoporosis as a disease where the bone mass density (BMD) falls within a 2.5 standard deviation or lower than the average value [11]. The greater part of fractures tends to happen between the−2.5 < *T* − score < −1 range [12]. A discrepancy in bone resorption and development is the means through which osteoporosis develops [13] and is caused by a decrease in bone-forming mature osteoblast populations. This decrease in osteoblast populations is caused by multiple factors such as mesenchymal stem cells (MSCs) altering their biology, osteoblast progenitors that lack proliferation ability, a rise in apoptosis, and an increase in the build-up of marrow adipocytes [14–16]. As it stands, it is estimated that osteoporosis affects two hundred million individuals, and approximately nine million fractures that occur per year are brought on by osteoporotic disease [17]. Osteoporosis is understood to be unbiased to a specific gender, race, or age; therefore, it has the ability to affect a variety of individuals [11]. However, this disease has shown a tendency of affecting Caucasians, elderly population groups, and specifically postmenopausal females [18]. Osteoporosis is problematic as it affects the quality of life of a patient through financial burden, increasing probable painful fractures as well as morbidity and mortalities [19]. Osteoporotic treatment currently remains limited as most treatments, which are drug-based therapies, create severe side effects such as osteonecrosis of the jaw and atypical femoral fractures [20, 21] and may raise breast cancer, stroke, and cerebral infarction risks if used for extended periods [22].

### 2.2. Bone Marrow Stromal Cells

Bone marrow stromal cells (BMSCs) comprise of a subset of stem cells known as MSCs, multipotent stromal cells, or skeletal stem cells, which are able to differentiate into osteoblasts and take part in an essential role within the “tissue engineering” of new bone [23]. Current studies have identified BMSCs, when featured in syngeneic, allogeneic, and xenogeneic situations, to exhibit immunoregulatory traits [24]. BMSCs are regarded as rejection tolerant supposed by their secretion of varying immunosuppressive factors and minimal MHC molecule expression; thus, a surge in immune reaction postinjection does not occur [25]. Despite the minimal MHC molecule expression, BMSCs are capable of NK cell induced lysis defence due to numerous mechanisms [26]. A majority of studies suggest that BMSCs stimulate the adaptive immunity in combination of production of their specific memory T cells [27] within a small period of time postinfusion into a host and disappear after approximately two to four days [24]. In summary, BMSCs secrete soluble factors that induce regulatory T cell and anti-inflammatory M2 monocyte/macrophage production. In company of these cells, BMSCs restrain helper T cells, B cells, NK cells, and mast cells' functions. Skeletal progenitors located within the bone marrow cavity are accountable for the formation of the 3-dimensional skeletal structure that provides a hematopoietic niche, due to osteoblasts, chondroblasts, adipocytes, and stromal fibroblasts' differentiation [28]. MSCs were originally designated over 25 years ago to describe a class of human and mammalian bone marrow and periosteum cells that could be isolated and grown in culture while preserving their *in vitro* potential to induce a range of mesodermal phenotypes and tissues [29]. These nonhematopoietic cells were first identified by Friedenstein et al. in the bone marrow, identified as a spindle in shape and displayed properties of stem cells [30, 31]. Following this discovery, MSCs were extracted from adipose [32], muscle [33], and umbilical cord [34] sources. Today, these cells are known to reside in a majority of tissues like the bone marrow, muscle, fat tissue, and liver [35]. MSCs are acknowledged as adult stem cells because of their multipotency and self-renewal abilities [36]. MSCs are only regarded as such if their appearance is fibroblast-like and are able to differentiate into either osteogenic, adipogenic, or chondrogenic cell lineages [37]. MSCs even possess a special subset of cells referred to as dental pulp stem cells (DPSCs) which display effortless stimulation in osteogenic differentiation [38], particularly desirable for dentistry applications. Both bone marrow-derived mesenchymal stem cells (BMDMSCs) and ADSCs have been the commonly studied and characterized cell sources [37]. BMDMSCs are located in the bone marrow and harvested through a bone marrow aspiration under a local sedation which yields low cell numbers and has a tendency to differentiate into osteogenic cell type [39]. BMDMSCs function foremost as osteogenic progenitors as well as regulate hematopoietic stem cell (HSC) function through the secretion of trophic factors and maintenance of the HSC niche [36]. ADSCs are located in adipose tissue and harvested from minimally invasive lipoaspirates which yield large cell amounts and are better able to differentiate into a variety of cell types particularly adipocytes, osteoblasts, and chondrocytes making these cells a preferred cell choice [39].

### 2.3. Osteoblasts

**Figure 1:**
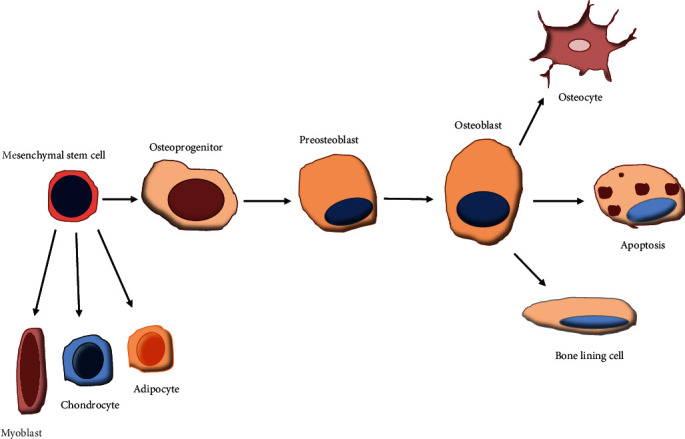
Osteogenic lineage. The process of osteogenic differentiation of MSCs.

The bone remodelling process consists of two significant processes, the one being bone resorption which is enabled by osteoclast cells [40] and the other being the development of new bone which is the responsibility of the osteoblast cells [41]. MSCs produce osteoblasts with the assistance of transcription factors like Osterix, runt-related transcription factor 2 (Runx2), octamer-binding transcription factor 3/4 (Oct4), and L-myc [42]. These significant functions of osteoblasts are to preserve and renew bone mass, control the quality of bone, and regulate overall skeletal performance [43–45]. Osteoblasts perform their significant functions by the creation and release of a variety of proteins needed for the formation of bone as well as the mineralization of the bone matrix [46]. Osteoblast performance is affected by a range of influences such as the interactions of the cellular matrix [47], transcriptional and epigenetic mechanisms [48], inflammatory activities [49], and cell to cell communication [50]. As displayed in Figure 1, in the beginning of osteogenesis, MSC populations will continuously proliferate until committed to osteoblasts as this then decreases the rate of their proliferation. During the matrix maturation phase, early osteoblasts will secrete osteogenic markers like alkaline phosphatase, followed by the mineralization phase, where late osteoblasts secrete osteocalcin. At the end of bone formation, these cells will either develop into bone lining cells and osteocytes or submit to apoptosis [40].

### 2.4. Regenerative Medicine

**Figure 2:**
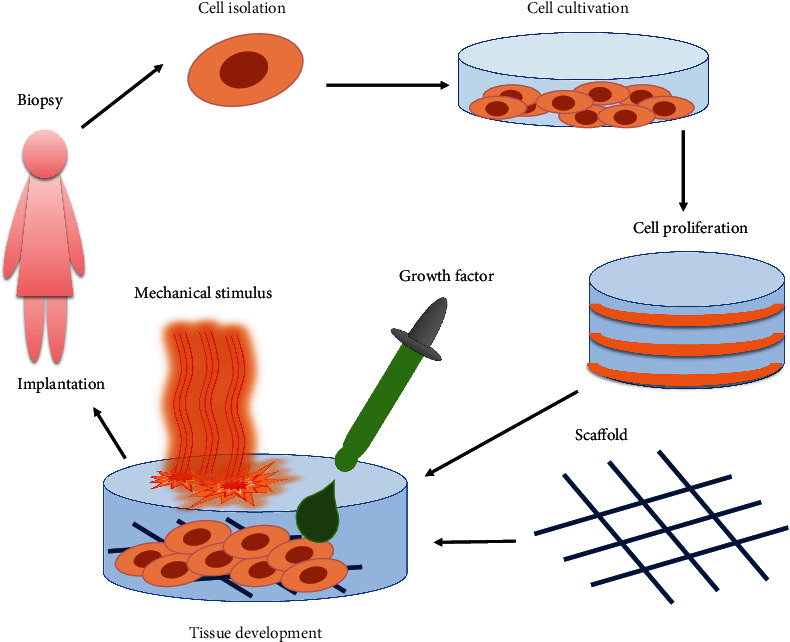
Regenerative medicine. The isolation of stem cells from the host through biopsy, which are encouraged to proliferate and differentiate using various growth factors and physical mechanisms. After differentiation, cells are transplanted back into the host.

Regenerative medicine is a developing field of medical science that is aimed at repairing the functionality and heal tissues or organs that have become diseased, injured, or affected by age-related complications [3]. Regenerative medicine emerged as a strategy to address the lack of available donor organs and tissues as well as critical immune rejection responses [51]. To influence the healing of tissue, restorative cells need to properly influence both the structure and function of new tissues. This is done using numerous combinations of both biological and chemical compounds and newly produced cells [52]; this is the basis of regenerative medicine (see Figure 2) [53].

The materials used for regenerative medicine are a significant aspect as they influence structure and function of the new tissue, offer growth factors, and control the behaviour of cells by imitating the extracellular matrix [54]. Recently approved Food and Drug Administration (FDA) regenerative medicine products have either improved or have remained similar to previous products [55]. This promotes both healing and regeneration; however, there still remains a gap in the treatment of disease [56]. Currently, stem cell therapy is taking the lead in the field of regenerative medicine because of SCs' remarkable ability to differentiate into multiple cell types and to unlimitedly self-renew [3].

### 2.5. Biomaterials/Scaffolds in Regenerative Therapy

Tissue engineering is an advancing tool for the regeneration of bone; the blend of cells, scaffolds, and biofactors often leads to a successful outcome. The scaffold is a bone tissue engineering (BTE) tool intended to trounce autografting and allografting shortcomings [57]; this is a 3D matrix designed for cells with an osteogenic affinity to attach and proliferate on the scaffold surface [58]. Two significant characteristics for a bone scaffold are to be (i) osteoconductive, the inner relocation of mesenchymal cells, osteoblasts, osteoclasts, and additional vasculature is to be offered by the scaffold, and (ii) osteoinductive, the stimulation of cells of different cell lineages to be differentiated into an osteogenic cell lineage [59]. Adult stem cells, particularly human bone marrow stem cells, are commonly used for BTE as the use of these cells entails no ethical conflict nor presents as a risk for immune rejection [60]. Scaffolds have been manufactured from a diverse assortment of materials such as bioceramics, biopolymers, metals, and composites [57]. These materials vary in factors like porosity architecture, mechanical ability, cell bondage, biocompatibility, cell proliferation, osteogenic differentiation ability, and mineralization, all of which influences the scaffold osteoconductivity and osteoinduction [57]. Additional studies established that osteogenesis is further enhanced by the combination of scaffolds and osteogenic growth factors such as fibroblast growth factor (FGF), insulin-like growth factor (IGF), epidermal growth factor (EPG), and bone morphogenic protein (BMP) which encourage bone manipulability [59].

### 2.6. Stem Cell Regenerative Therapy

Stem cells are deemed as a significantly unique tool for regenerative medicine because of their self-renewal and multidifferential characteristics [4]. The ability of cells to abundantly divide while preserving their undifferentiated state is called self-renewal [61]. This ensures that stem cells are increased during development; during adulthood, the cell numbers remain constant and posttrauma, these cell numbers are brought back to the normal amount [61, 62]. Stem cells do not self-renew to a large extent when under physiological states, but when cell injury calls for regeneration, then stem cell potentials alter depending on the environment of these physiological changes [62]. The potential of a stem cell to differentiate into various types of cells is termed as potency [61]. The tissue from which stem cells are extracted will determine their potency because this changes the differentiation potential [4]. Stem cells are grouped according to their differentiation potentials as totipotent stem cells that form when a sperm fertilizes an oocyte to produce a zygote, possessing the ability to differentiate into embryonic and extraembryonic cell types [63]; pluripotent stem cells differentiate into the three germ layers: the endoderm, the mesoderm, and the ectoderm; examples of these cells are embryonic stem cells (ESCs) and induced pluripotent stem cells (iPSCs) [64]; multipotent stem cells produce various specialized cells of a particular lineage [65], and unipotent cells can only differentiate into one cell type but are able to self-renew [61]. Stem cells are divided into the following types: ESCs, iPSCs, tissue-specific progenitor stem cells (TSPSCs), umbilical cord stem cells (UCSCs), and MSCs [66]. ESCs yield large cell amounts through indefinite division and differentiate into multiple types of cells [63, 67]. Despite these cells being an ideal regenerative cell type, the use of these cells come with immense ethical concerns because their isolation requires the destruction of embryos during their blastocyst-stage [68]. iPSCs are appealing cells because they are made from the cells of the patient which overcomes ethical challenges as well as cell transplant rejections [69, 70]. However, the differences between ESCs and iPSCs remain unclear as differences in gene expression, DNA methylation, and donor cell epigenetic memories are because of induction and culture condition variations [64]. TSPSCs are not a preferred cell source because their cell population amount to the total population of cells is insufficient, therefore, rendering them unsatisfactory for harvesting [71]. UCSCs originate from the umbilical cord, which is rich in HSCs and MSCs, and make an ideal cell source because they are obtained noninvasively and unlike ESCs; their use has minimal ethical conflicts [72]. MSCs are multilineage cells primarily located in the bone marrow [73, 74] highly capable of self-renewal, differentiation, and proliferation [36]. MSCs have the ability to differentiate into either osteogenic, adipogenic, or chondrogenic cell lineages, as well as differentiate into tenocytes, smooth muscle cells, and stromal cells [37].

MSCs further derive into two cell types. The first cell type is BMDMSCs located in the bone marrow and functions as osteogenic progenitors and regulators of the HSC niche [36]. However, their extraction process is extremely invasive and their proliferation and differentiation abilities are weaker in comparison to other cell types which makes them unlikely for selection as a cell source [75]. The second type is ADSCs; these cells are easily isolated from adipose tissue via a harmless lipoaspirate [53, 76] and provide an abundance of cells able to self-renew. ADSCs are able to differentiate into multiple cell types such as adipocytes, osteoblasts, chondrocytes, and smooth muscle cells [39].

### 2.7. Adipose-Derived Stem Cells

ADSCs prove to be a preferable cell source because of two significant qualities: these cells are easily sourced from adipose tissues preferably located at the hip and abdomen in abundant amounts [39, 77] and have no ethical concerns like those of ESCs because ADSCs are isolated from autologous fat [78]. Other advantageous qualities include antiapoptotic, immunomodulatory, anti-inflammatory, and antiscarring [79]. ADSCs are harvested in one of three ways: Coleman's technique [80], liposuction [81], and excision [80], all of which are less invasive than that of harvesting ESCs. The differentiation potentials of ADSCs do not change depending on the method used [39] although, as with any invasive procedure, there lie associated risks such as bleeding, infection, necrosis, and injuries to nerves [36]. In comparison to BMDMSCs, ADSCs are more useful for the making of collagen than osteogenesis. Nonetheless, ADSCs are consistent for extended periods of time in culture, both morphologically and genetically, and as a result are better able to proliferate [82] and yield abundant amounts of cells during harvesting. Thus, ADSCs are selected as the preferable cell source [36]. Unfortunately, there is a lack of consensus regarding whether or not the age of the patient influences the ADSC properties; some studies state that both the quality and proliferation of the cells have no association with age [83] whereas others state that patients younger in age display increased osteogenic and angiogenic abilities and that older patients display lower differentiation and proliferation abilities [39].

### 2.8. Differentiation of ADSCs into Osteoblasts

To achieve successful differentiation of osteogenic lineages from ADMSCs *in vitro*, these cells need to be cultured in Dulbecco's Modified Eagle's Medium (DMEM) [84] with a combination of growth factors like ascorbic acid, *β*-glycerol phosphate, dexamethasone, and 1,25 vitamin D3 [85]. Additional factors like bone morphogenic protein 2 (BMP-2) further stimulate osteogenic differentiation of these cells [86]. Upon successful differentiation, the ADSCs exposed to these factors in culture will produce osteoblastic genes and proteins such as alkaline phosphatase, osteonectin, BMP-2, osteopontin, BMP-4, type 1 collagen, and Runx2 [87–89]. The successful osteogenic production from ADSCs indicates that these cells are capable of migration, proliferation, and differentiation should *in vivo* transplantation take place, therefore, promising the regeneration of the targeted bone tissue [90–92]. However, consistent success in all studies with the ADSC potential to differentiate into osteogenic lineages and proliferate still requires further investigation both *in vitro* and *in vivo* [93].

#### 2.8.1. Biological Differentiation

A biological growth factor is a material that naturally occurs and is able to promote the proliferation and differentiation of a particular, desired cell type. In order for ADSC to differentiate into osteogenic cell lines, various biological growth factors such as insulin-like growth factor-1 (IGF-1), BMP-2, Wnt, basic fibroblast growth factor (bFGF), ascorbic acid, and 1,25 vitamin D3 [94] are added for the acceleration of proliferation, differentiation, and regulation of osteoblast cells as seen in Table 1: TGF-*β*1, insulin, transferrin, dexamethasone, and ascorbic acid for chondrogenic differentiation; insulin, transferrin, and selenium for skeletal myogenic differentiation; and dexamethasone, ascorbic acid, and *β*-glycerophosphate for osteogenic differentiation. Therefore, the same MSC population in the mesoderm exposed to different extrinsic stimuli can initiate differentiation towards a specific cell type by triggering a tissue-specific transcription factor, such as SOX5/6/9 for chondrocytes and Runx2/Osterix for osteoblasts [95]. Significantly, some growth factors desirably inhibit the differentiation of ADSCs [96] such as epidermal growth factor (EFG) [97], platelet-derived growth factor (PDGF) [98], and vascular endothelial growth factor (VEGF) [99].

#### 2.8.2. Chemical Differentiation

Chemical growth factors are used in addition to biological growth factors due to their regulatory capacity to ensure the expected fate of MSC differentiation [108]. Often, factors such as dexamethasone, calcium phosphate families, hypoxia-inducible factor, and beta-glycerol phosphate (see Table 2) are added for osteogenic differentiation purposes and to prevent adipogenesis from taking place instead [85].

Although it has been established that certain growth factors, biological or chemical, have the potential to induce differentiation of ADSCs into osteoblast-like cells, it has also been noted that the control of osteogenesis and adipogenesis in ADSCs is closely related. ADSCs have a preferential commitment to adipogenic lineages unless specifically controlled [112]. This concern for control of lineage-specific differentiation using a combination of growth factors has prompted the use of combining mechanical stimulation for differentiation of ADSCs into osteoblasts. One such method is the use of PBM, where numerous studies, using either PBM therapy or in combination with biomaterials [113], have significantly sped up the synthesis of the bone matrix by increasing vascularization and decreasing inflammatory responses [114], which raises the osteocyte populations as well as bFGF [115] along with promoting proliferation of cells [116].

#### 2.8.3. Biophysical Differentiation

Within the SC microenvironment, MSCs are exposed to an assortment of biophysical cues. For example, hydrostatic pressure, fluid flow and accompanying shear stress, substrate strain and stiffness, substrate topography, and electromagnetic fields are all biophysical indicators responsible for cell membrane morphology changes and cell-matrix contacts and intracellular junction force generation producing intracellular stress [117]. The significance of biophysical prompts is identified to stimulate gene expression changes bringing about SC differentiation as identified with *in vitro* osteoblastic differentiation amongst BMSCs [118]. However, a recent study identified ADSCs for the purpose of osteogenic differentiation in the presence of prefabricated scaffolds, as a more feasible cell source than BMSCs and produced a successful bone regenerative outcome [119]. Studies have identified the use of mechanical stimulants like PBM to facilitate the proliferation and differentiation of various cell lines [120], and therefore, these might be a viable biophysical differentiation source to use for ADSC differentiation into osteogenic lineages.

### 2.9. Photobiomodulation

When light is used through coherent or incoherent light sources in a visible and near-infrared (NIR) range, this is termed as PBM which stimulates endogenous chromophores bringing about both photochemical and photophysical reactions [120, 121]. Even though the process is still not fully comprehended, it is understood that cell signalling cascades, as well as effector molecules, are stimulated, promoting cell performance alterations [120, 122]. A commonly proposed biochemical reaction of PBM using wavelengths between 600 and 1100 nm is the “Cytochrome c Oxidase (CCO) Theory.” This theorem is based on the penetration of red or NIR light through a cell's membrane, targeting its mitochondria and initiating light absorption by cytochrome c located within the mitochondria [123]. This enzymatic chromophore then aids in the electron transport chain during ATP production. An increase in ATP amounts tends to induce an increase in gene transcription within the cell nucleus bringing about an increase in DNA and RNA synthesis initiating cell proliferation [124]. .Currently, an ideal method to successfully increase proliferation and facilitate differentiation of stem cells through PBM is still being explored for clinical use [116, 125, 126]. The cellular mechanism variations caused by photochemical procedures, dependency of dose [127], cell line limitations for dosage [128], and the number of times as well as the period between each exposure [129] remain under investigation for the establishment of PBM parameters. However, the consensus amongst research is that PBM stimulates proliferation of cells when using a wavelength of/between 660 and 850 nm and fluence of/between 5 and 10 J/cm^2^ [130]. Additionally, green light PBM ranging from 495 nm to 570 nm has been identified to better improve cell differentiation; however, the biochemical mechanism of this wavelength and further successful differentiation ability remain under investigation [116, 125, 126]. Notably, green light is anticipated to increase intracellular ROS in succinct amounts during the use of a low fluency which has demonstrated enhanced involvement in cell differentiation [125]. However, studies which used high fluences, greater than 10 J/cm^2^, identified a biphasic dose response which expressed significantly increased ROS levels, cell damage, and cell death [131].

### 2.10. Effects of PBM on ADSCs

According to treatment responses, ADSCs when irradiated at a wavelength of 825 nm with fluences ranging between 5 and 15 J/cm^2^ are stimulated but will often be inhibited by the use of a higher fluence of 20 J/cm^2^ [132]. A study using a low-power laser with the parameters of 660 nm ± 20 nm, 6 J/cm^2^, and 10 mV/cm^2^ on ADSCs increases angiogenic factors and decreases apoptosis occurrence [133]. Another study using a low-power laser with the parameters of a wavelength of 660 nm + 20 nm, 220 V + 22 V, and 50 Hz regulates the adhesion of cells and their migration signals by increasing EKK1/2 and FAK thus increasing overall cell migration. An increase in the proliferation, viability, and growth factors, particularly hepatocyte growth factor (HGF) and PDGF, was demonstrated in this study [134]. A study that used a low-power laser of 808 nm, 3 J/cm^2^, and 0.2 W/cm^2^ showed both an increase in cell proliferation and viability [135]. Significantly, a low-power laser of 660 nm and 0.5 and 1 J/cm^2^ with dose dependency affects ADSCs and BMDMSCs by increasing both cellular growth and proliferation without making nuclear modifications and secretes growth factors VEGF, HGF, and FGF [136]. The studies of significantly producible results have been summarized in Table 3.

### 2.11. Combined Effects of PBM and Differentiation Inducers on ADSCs

The combination of PBM, specifically green light, red light, and NIR wavelengths, with the addition of multiple growth factors, is believed to facilitate both cellular activity regulation and the differentiation of ADSCs [140]. The potential stimulation and inhibition consequences of PBM have on ADSCs tend to be wavelength and fluency factor dependent [120]. There have been numerous studies performed using various PBM parameters in combination with ADSC differentiation inducers such as dexamethasone, ascorbic acid, beta-glycerophosphate, L-glutamine, and ascorbate-2-phosphate; a few examples of these studies are as follows (see Table 4). A study differentiating ADSCs into osteoblasts using various wavelengths but the same 3 J/cm^2^ dose five times every second day established osteoblast differentiation stimulation to be successful with 420 nm and 540 nm wavelengths [125]. Another study that used MSCs at a wavelength of 635 nm had no change in cell viability but a wavelength of 808 nm increased the deposits of calcium thus impacting osteogenic differentiation [141]. The differentiation of ADSCs into osteogenic cell lines was also seen to be enhanced by the use of NIR laser light [142]. Another report stated that ADSCs were proliferated and differentiated by PBM at a red of 660 nm and a NIR of 810 nm wavelengths and it is understood that mitochondrial activity as well as the production of ATP is stimulated by PBM at this particular NIR wavelength [143]. Despite the majority of studies displaying positive photobiostimulatory outcomes when regenerating bone using low-level laser therapy, standardized parameters are yet to be established for reproducible results [130].

### 2.12. Current and Future Challenges

The current rise in global concern for osteoporosis as well as the risky, long-term treatment solutions for this disease has sparked the search and development of an efficient and minimally harmful long-term treatment [144]. The promising branch of regenerative medicine, SC therapy, is the emerging, probable solution to this increasing concern [53]. The principle of regenerative medicine is based on the healing of injured tissue via cell usage and combinations of various biological and/or chemical growth factors to restore cell structure, cell functionality, and create newly formed repaired tissues [145]. SC therapy is the rising star of regenerative medicine as SCs are uniquely able to infinitely self-renew and possess a multipotent ability into ESCs, iPSCs, TSPSCs, UCSCs, or MSCs cell types, where each cell type provides its own benefits and limitations [66]. ADSCs are obtained from a minimally invasive harvest and hold a large cell yield [5, 78]. The nonethically conflicting ADSCs significantly possess the ability to differentiate into adipocyte, osteoblast, and chondrocyte cell lineages [39]. According to studies, the differentiation of ADSCs into a specific cell lineage requires the influence of various growth factor combinations [146]. Successful *in vitro* ADSC differentiation into an osteogenic cell lineage has been accomplished by the unaccompanied use of PBM using biological and/or chemical growth factors such as ascorbic acid, *β*-glycerol phosphate, dexamethasone, 1,25 vitamin D3 [85], and BMP-2 [86] supplemented in an induction medium. The use of a specific concentration as well as the combination of these growth factors varies amongst studies and requires optimization for result reproducibility purposes along with functionality testing, as ADSCs have a tendency of favouring differentiation into adipogenic cell lineages unless purposely influenced into a different cell line [112]. PBM alone is understood to only be involved in biostimulation where an increase in cell proliferation and viability is noted when using red or NIR light [147]. Green PBM also has a biostimulatory effect causing an increase in intracellular ROS [125] aiding in the preparation of ADSC differentiation. However, it can therefore be said that the combined use of transducers and PBM on ADSCs for differentiating into osteogenic cell lineages could be a more effective technique than using these factors alone, where *in vitro* optimization and successful differentiation of ADSCs can be then studied *in vivo*. According to studies above, PBM at wavelengths ranging from 660 nm to 850 nm and fluences ranging between 5 and 15 J/cm^2^ have facilitated ADSC differentiation into a desired cell lineage and enhanced proliferation [142]. Ideal ADSC differentiation into osteogenic cell lineage occurrence may be deduced from the above at wavelengths of either red light, green light, or NIR at a fluence below 15 J/cm^2^. The use of green light wavelengths has been recognized to stimulate calcium ion channel exchange that leads to the increased expression of intracellular ROS in physiologically viable amounts which is understood to enhance cell differentiation [125]. The use of red light or NIR wavelengths has displayed efficient cytochrome c absorbance which enhances cell proliferation as well as cell viability and, when in the presence of differentiation transducers, facilitates the differentiation of cells [110, 126]. A fluency that is greater than 10 J/cm^2^ initiates biphasic dosing and ultimately causes cell death [131]; thus, fluences below 10 J/cm^2^ are favorable. A power output below 100 mW will exclude the thermal effect [148] that can be introduced with light exposure omitting this external factor that might influence the cells negatively. The usage of these parameters will be best fitting for combining PBM with the use of differentiation growth factors to effectively enhance the differentiation of ADSCs into osteogenic cell lineages. The parameters of PBM such as the wavelength of the laser, the energy fluence, the number of times of exposure, and the period between each exposure [120] remain under current *in vitro* investigation for the establishment of optimal PBM parameters and a fixed protocol. The development of a fixed *in vitro* protocol for the use of ADSC differentiation into osteoblasts will produce a safe and sound procedure that may be efficiently translated *in vivo* for the clinical use of osteoporotic treatment and as a regenerative tool [130].

## 3. Conclusion

In conclusion, the use of various biological and chemical growth factors, in combination with a physical inducer, particularly PBM successfully proliferates and differentiates ADSCs into osteoblast cells. However, this procedure awaits the establishment of an ideal, set protocol of assured growth factors and PBM parameters as there are various published papers debating factors such as growth factors, wavelengths, and fluencies. The established protocol would ensure consecutive successful proliferation and differentiation for the specific ADSC cell lines into osteoblasts. Despite these inconsistencies, the use of PBM especially on this cell line for osteoblastic differentiation has been remarkable and remains promising to move forward in methodology, to achieve success *in vitro*, and possibly to achieve clinical studies as a possible form of treatment for osteoporosis.

## Figures and Tables

**Table 1:** Biological growth factors introduced to ADSCs for osteogenic differentiation.

Biological growth factors	Outcome of growth factors	Refs
Insulin-like growth factor 1/2	Stimulate both proliferation and terminal differentiation of MSCs, fine-tuning transcription factor expression levels and activity, and defining commitment towards specific lineages from the three germ layers. Overall, IGF-1 and IGF-2 play a significant role in MSC osteogenic differentiation and bone health.	[95, 100]
IGF-1	IGF-1 expression in MSCs enhances their proliferation with lower apoptosis; overexpression of IGF-1 in osteoblasts can accelerate the rate of bone formation and increase the pace of matrix mineralization, IGF-1-transfected human MSCs were able to upregulate expression of various osteoblast genes. 100 ng/mL of IGF-1 promotes differentiation into osteoblast-like cells.	[101–104]
IGF-2	IGF-2 appears to be more prominent than IGF-1 in promoting MSC self-renewal.	[100]
BMP-2	Accelerates proliferation and differentiation of osteoblast cells.	[105]
Wnt3a	Increased cell numbers and expanded the pool of MSCs capable of colony-forming unit- (CFU-) fibroblast (CFU-F) and CFU- osteoblast (CFU-O); regulates osteoblast differentiation and maturation.	[106]
Wnt5a	Maintained cell numbers and CFU-F and CFU-O numbers and increased the number of CFU-O.	[106]
bFGF	bFGF was shown to be required in maintaining stemness and proliferation. Enhances the development of osteogenic cells.	[94, 107]
Ascorbic acid	Proliferates differentiated osteoblasts and inhibition of ADSC differentiation.	[96–99]
1,25 Di-hydroxy vitamin D3	Proliferates differentiated osteoblasts and inhibits ADSC differentiation.	[96–99]

**Table 2:** Chemical growth factors introduced to ADSCs for osteogenic differentiation.

Chemical growth factors	Outcome of growth factors	Refs
Dexamethasone	Proliferates differentiated osteoblasts and inhibits ADSC differentiation.	[85, 108, 109]
Calcium phosphate	Promotes the differentiation of osteogenic cell lines.	[110]
Hypoxia-inducible factor	Regulates osteogenesis.	[111]
Beta-glycerol phosphate	Proliferates differentiated osteoblasts and inhibits ADSC differentiation.	[85, 108, 109]

**Table 3:** Effects of PBM on ADSCs.

Wavelength (nm)	Output power (mW)	Energy density (J/cm^2^)	Irradiation (mW/cm^2^)	Effects	Refs
660	30	0.2	1.07	The viability of cells was increased.	[137]
650	523	2, 4, 8	6.67	The 4 J/cm^2^ enhanced ADSC proliferation.	[138]
680	3 and 4.5	—	—	Migration was sped up.	[134]
808	200	3	0.2	Proliferation was sped up.	[135]
636	85	5	9.3	Both the cell viability and proliferation were increased.	[139]
825	—	5, 10, 15, 20	—	ADSC biphasic dose response occurred with fluences 15 and 20 J/cm^2^.	[132]
660 ± 20	—	6	10	An increase in angiogenic factors and decreased apoptosis.	[133]
660	30	0.5, 1.0	—	Increased cellular growth and proliferation as well as VEGF, HGF, and FGF growth factor secretion.	[136]

**Table 4:** The combined effects of using PBM and differentiation inducers for MSCs into osteoblasts.

Differentiation inducers	Laser parameters		Effects	Refs
	Wavelength (nm)	Fluency (J/cm^2^)		
Dexamethasone, ascorbic acid, beta-glycerophosphate	420, 540	3	Increased the concentration of intracellular calcium thus increased osteogenic relative gene expression.	[125]
Dexamethasone, ascorbic acid, beta-glycerophosphate, L-glutamine	635, 808	0.4	Increased the focal adhesion-localized vinculin which promotes osteogenic differentiation. Osteogenic differentiation was encouraged.	[141]
Dexamethasone, beta-glycerol phosphate, ascorbate-2-phosphate	809	0.5, 1, 2	Mineralization occurred which indicated cell differentiation.	[142]
Dexamethasone, ascorbic acid, beta-glycerophosphate	810, 980	3, 0.3	Osteogenic-related gene expression was upregulated.	[143]

## Data Availability

The quantitative and qualitative data supporting this systematic review are from previously reported studies and datasets, which have been cited. These prior studies (and datasets) are cited at relevant places within the text as references.
